# Chromosome-level assembly of *Dictyophora rubrovolvata* genome using third-generation DNA sequencing and Hi-C analysis

**DOI:** 10.1093/g3journal/jkad102

**Published:** 2023-05-13

**Authors:** Lu Ma, Chi Yang, Donglai Xiao, Xiaoyu Liu, Xiaoling Jiang, Hui Lin, Zhenghe Ying, Yanquan Lin

**Affiliations:** Institute of Edible Mushroom, Fujian Academy of Agricultural Sciences, Fuzhou 350012, China; National and Local Joint Engineering Research Center for Breeding and Cultivation of Featured Edible Muhsroom, Fujian Academy of Agricultural Sciences, Fuzhou 350012, China; Institute of Edible Mushroom, Fujian Academy of Agricultural Sciences, Fuzhou 350012, China; National and Local Joint Engineering Research Center for Breeding and Cultivation of Featured Edible Muhsroom, Fujian Academy of Agricultural Sciences, Fuzhou 350012, China; Institute of Edible Mushroom, Fujian Academy of Agricultural Sciences, Fuzhou 350012, China; National and Local Joint Engineering Research Center for Breeding and Cultivation of Featured Edible Muhsroom, Fujian Academy of Agricultural Sciences, Fuzhou 350012, China; Institute of Edible Mushroom, Fujian Academy of Agricultural Sciences, Fuzhou 350012, China; National and Local Joint Engineering Research Center for Breeding and Cultivation of Featured Edible Muhsroom, Fujian Academy of Agricultural Sciences, Fuzhou 350012, China; Institute of Edible Mushroom, Fujian Academy of Agricultural Sciences, Fuzhou 350012, China; National and Local Joint Engineering Research Center for Breeding and Cultivation of Featured Edible Muhsroom, Fujian Academy of Agricultural Sciences, Fuzhou 350012, China; Institute of Edible Mushroom, Fujian Academy of Agricultural Sciences, Fuzhou 350012, China; National and Local Joint Engineering Research Center for Breeding and Cultivation of Featured Edible Muhsroom, Fujian Academy of Agricultural Sciences, Fuzhou 350012, China; Institute of Edible Mushroom, Fujian Academy of Agricultural Sciences, Fuzhou 350012, China; National and Local Joint Engineering Research Center for Breeding and Cultivation of Featured Edible Muhsroom, Fujian Academy of Agricultural Sciences, Fuzhou 350012, China; Institute of Edible Mushroom, Fujian Academy of Agricultural Sciences, Fuzhou 350012, China; National and Local Joint Engineering Research Center for Breeding and Cultivation of Featured Edible Muhsroom, Fujian Academy of Agricultural Sciences, Fuzhou 350012, China

**Keywords:** *Dictyophora rubrovolvata*, whole-genome sequencing, functional annotation, lignocellulose degradation, CAZymes

## Abstract

*Dictyophora rubrovolvata*, a rare edible mushroom with both nutritional and medicinal values, was regarded as the “queen of the mushroom” for its attractive appearance. *Dictyophora rubrovolvata* has been widely cultivated in China in recent years, and many researchers were focusing on its nutrition, culture condition, and artificial cultivation. Due to a lack of genomic information, research on bioactive substances, cross breeding, lignocellulose degradation, and molecular biology is limited. In this study, we report a chromosome-level reference genome of *D. rubrovolvata* using the PacBio single-molecule real-time-sequencing technique and high-throughput chromosome conformation capture (Hi-C) technologies. A total of 1.83 Gb circular consensus sequencing reads representing ∼983.34 coverage of the *D. rubrovolvata* genome were generated. The final genome was assembled into 136 contigs with a total length of 32.89 Mb. The scaffold and contig N50 length were 2.71 and 2.48 Mb, respectively. After chromosome-level scaffolding, 11 chromosomes with a total length of 28.24 Mb were constructed. Genome annotation further revealed that 9.86% of the genome was composed of repetitive sequences, and a total of 508 noncoding RNA (rRNA: 329, tRNA: 150, ncRNA: 29) were annotated. In addition, 9,725 protein-coding genes were predicted, among which 8,830 (90.79%) genes were predicted using homology or RNA-seq. Benchmarking Universal Single-Copy Orthologs results further revealed that there were 80.34% complete single-copy fungal orthologs. In this study, a total of 360 genes were annotated as belonging to the carbohydrate-active enzymes family. Further analysis also predicted 425 cytochromes P450 genes, which can be classified into 41 families. This highly accurate, chromosome-level reference genome of *D. rubrovolvata* will provide essential genomic information for understanding the molecular mechanism in its fruiting body formation during morphological development and facilitate the exploitation of medicinal compounds produced by this mushroom.

## Introduction

*Dictyophora rubrovolvata*, a saprophytic fungus belonging to the family Phallaceae (Fu *et al.* 2019), is a widely artificially cultivated edible mushroom in Southwest China. *Dictyophora rubrovolvata* is also called “hong tuo zhu sun” (red-volva basket stinkhorn) in Chinese and regarded as the “queen of the mushroom” for its attractive appearance (Liao *et al.* 2015). *Dictyophora rubrovolvata* grows on the wet roots of bamboo groves or in the humus of bitter bamboo forests in Guizhou, Yunnan, and Sichuan provinces of China (Hang *et al.* 2012). It was initially discovered in Yunnan province of China in 1976 and then successfully artificially cultivated in 1983 (Zang and Ji 1985). In the cultivation cycle of *D. rubrovolvata*, the development process can be divided into 5 major stages: undifferentiated mycelia, primordia, ball-shaped stage, peach-shaped stage, and the mature stage (Wang *et al.* 2020). The mature fruiting body of *D. rubrovolvata* possesses a unique appearance, including a red-volva, white stipe, and white net-like veil.

*Dictyophora rubrovolvata* has been widely used as a functional food in daily life in China and Japan for its variety of nutrients, including proteins, amino acids, minerals, vitamins, thiamine, riboflavin, nicotinic acid, and polysaccharides (Deng *et al.* 2016). *Dictyophora rubrovolvata* has also been reported to have many biological and pharmacologic activities, such as antiaging and hypoglycemic (Ye, Wen, Peng, *et al.* 2016), antifatigue and hypoxia endurance (Ye, Wen, Huan, *et al.* 2016), etc. Up to now, *D. rubrovolvata* was still considered a rare edible mushroom in China. However, compared with other mushrooms, such as *Flammulina velutipes*, *Pleurotus eryngii*, and *Hypsizygus marmoreus*, its biological and genetic information remains limited, which impedes the breeding of high-quality cultivars.

In recent years, the genomes of many basidiomycetes have been obtained, including *Stropharia rugosoannulata* (Li *et al.* 2022), *Naematelia aurantialba* (Sun *et al.* 2021), *Sparassis latifolia* (Xiao *et al.* 2018), *F. velutipes* (Park *et al.* 2014), *P. eryngii* (Dai *et al.* 2019), *H. marmoreus* (Jin Jing *et al.* 2015), *Lentinula edodes* (Shim *et al.* 2016), and *Agaricus bisporus* (Morin *et al.* 2012). The availability of these increased genome sequences promotes research on the development and utilization of medical and pharmaceutical products. Hi-C technology provides a unique and powerful tool to study nuclear organization chromosome architecture (Belton *et al.* 2012). In recent years, the Hi-C technique has been used to assemble high-quality genomes for many mushrooms. An updated draft genome sequence of *S. latifolia* was generated by Oxford Nanopore sequencing and the Hi-C technique, a 41.41-Mb chromosome-level reference genome of *S. latifolia* was assembled, and 13,103 protein-coding genes were annotated (Yang *et al.* 2021). Based on the genome assembly obtained from second- and third-generation sequencing and Hi-C data, *Ophiocordyceps sinensis* strain 1,229 was found to possess 6 chromosomes with a strong telomere interaction between chromosomes (Meng *et al.* 2021). The Hi-C technique was also used to construct the chromosome-level genome in *L. edodes* (Gao *et al.* 2022; Yu *et al.* 2022) and *Ganoderma lucidum* (Wu *et al.* 2022).

In the present study, we used the PacBio Sequel II platform to sequence the *D. rubrovolvata* genome, and assembled a high-quality chromosome-scale reference genome using the Illumina platform combined with Hi-C scaffolding. The *D. rubrovolvata* genome sequence will be helpful for understanding the molecular mechanisms and advancing our understanding of its genetics and evolution.

## Materials and methods

### Fungal strains and strain culture

The *D. rubrovolvata* fruiting body was collected from Fuzhou, Fujian Province, China. The *D. rubrovolvata* strain Di001 was obtained from the fruiting body by tissue isolation. At present, this strain has been preserved at the Institute of Edible Mushroom, Fujian Academy of Agricultural Sciences. The strain was maintained on potato dextrose peptone agar slants and subcultured every 3 months.

### Extraction of genome DNA

To obtain sufficient cell amounts for genetic DNA extraction, *D. rubrovolvata* Di001 was inoculated into potato dextrose broth medium in a Petri dish for 10–15 days, and then the aerial mycelium was scratched out of the medium by sterilized cover glass. The sodium dodecyl sulfate method was used to extract the genomic DNA (Jiang *et al.* 2021). The genomic DNA concentration was determined using the Nanodrop spectrophotometer (Thermo Fisher Scientific, NANODROP2000) and Qubit Fluorometer (Invitrogen, Qubit 3 Fluorometer), and agarose gel electrophoresis was performed to check its integrity.

### De novo sequencing

The 15-kb SMRTbell library was constructed using the SMRTbell Express Template Prep Kit (version 2.0). The 350-bp small, fragmented library was constructed using the NEBNext ultra DNA library prep kit. After the library was qualified, the whole genome of *D. rubrovolvata* Di001 was sequenced using the PacBio Sequel II platform and Illumina NovaSeq PE150 at the Biomarker Technologies Corporation (Beijing, China), and the sequencing results were used for gene annotation.

### Genome assembly and assessment

To obtain chromosome-level whole-genome assembly for *D. rubrovolvata*, we utilized a combined approach of Illumina, PacBio and Hi-C technology for the genome assembly, and chromosome-level scaffolding.

Regarding the PacBio Sequel II platform, on the basis of removing the low-quality reads (<500 bp) from the raw data, the automatic error correction function of the single-molecule real-time (SMRT) portal software was used to further improve the accuracy of the seed sequences, and finally, the variant caller module of the SMRT link v5.0.1 software was used to correct and count the variant sites in the initial assembly results using the arrow algorithm (Berlin *et al.* 2015). The ccs (circular consensus sequencing) reads were assembled using Hifiasm v0.12 (https://github.com/chhylp123/hifiasm; Cheng *et al.* 2021), Pilon software (Walker *et al.* 2014) was used to further correct the assembled genome using the second-generation data, and finally, the genomes with higher accuracy was obtained. Regarding the Illumina NavaSeq PE150 platform, the clean reads were mapped to the *D. rubrovolvata* Di001 genome using Burrows–Wheeler Aligner software under its default parameters.

Benchmarking Universal Single-Copy Orthologs (BUSCO) v 2.0 software was used to assess the completeness of the genome assembly. The lineage data set of BUSCO was fungi_odb9 (number of species: 85; number of BUSCOs: 290).

### Hi-C library construction and assembly of the chromosome

Hi-C libraries were prepared as previously reported (Yang *et al.* 2021). Briefly, the sample was fixed with formaldehyde to maintain the 3D structure of DNA in cells and the restriction enzyme Hind III was applied to DNA digestion. Then, biotin-labeled bases were introduced using the DNA terminal repair mechanism. DNA was fragmented by a Covaris S220 focused ultrasonicator, and 300–700 bp fragments were recovered. The DNA fragments containing interaction relationships were captured by streptavidin immunomagnetic beads for library construction. Library concentration and insert size were determined using the Qubit 2.0 and Agilent 2100, respectively, and Q-PCR was used to estimate the effective concentration of the library. High-quality Hi-C libraries were sequenced on the Illumina NovaSeq PE150 sequencing platform, and the sequencing data were used for chromosome-level assembly (He *et al.* 2022). Hi-C data were filtered and evaluated using HiC-Pro software (Servant *et al.* 2015), it could identify the valid interaction pairs and invalid interaction pairs in the Hi-C sequencing results by analyzing the comparison results, and realize the quality assessment of the Hi-C libraries. The order and direction of scaffolds/contigs were clustered into super scaffolds using LACHESIS (Burton *et al.* 2013), based on the relationships among valid reads.

### Genome component prediction

*Dictyophora rubrovolvata* repeat sequence database was constructed by LTR_FINDER v1.05 (Xu and Wang 2007), MITE-Hunter (Han and Wessler 2010), RepeatScout v1.0.5 (Price *et al.* 2005), and PILER-DF v2.4 (Edgar and Myers 2005) based on structural and de novo prediction principles. PASTEClassifier (Wicker *et al.* 2007) was used to classify the database and then merged with Repbase (Bao *et al.* 2015) database to generate the final repeat sequence database. Finally, the RepeatMasker v4.0.6 (https://www.repeatmasker.org/RepeatMasker/; Tarailo-Graovac and Chen 2009) was used to predict the repeat sequence based on the built repeat sequence database.

The combination of ab initio–based prediction, RNA-seq-based prediction, and homology-based prediction was used to predict protein-coding genes. For ab initio–based prediction, Augustus v2.4 (Stanke and Waack 2003), Genscan (Burge and Karlin 1997), GeneID v1.4 (Alioto *et al.* 2018), GlimmerHMM v3.0.4 (Majoros *et al.* 2004), and SNAP (version 2006-07-28; Korf 2004) were employed under their default parameters. The GeMoMa v1.3.1 (Keilwagen *et al.* 2016) was used for homology-based prediction. For RNA-seq-based prediction, Hisat2 v2.0.4 (Pertea *et al.* 2016) and Stringtie v1.2.3 (Pertea *et al.* 2016) were employed based on reference transcripts (NCBI SRR23948425). TransDecoder v2.0 (https://github.com/TransDecoder/TransDecoder/wiki; The Broad Institute, Cambridge, MA, USA) and PASA v2.0.2 (Campbell *et al.* 2006) were used for unigene sequence prediction. Finally, the results were integrated by using EVM v1.1.1 (Haas *et al.* 2008) and PASA v2.0.2 (Campbell *et al.* 2006) to predict all protein-coding genes.

### Functional annotation

The predicted proteins were blasted (*e*-value: 1e−5) against nonredundant protein sequence (Nr), Swiss-Prot, TrEMBL, Kyoto Encyclopedia of Genes and Genomes (KEGG), and Clusters of Orthologous Groups (KOG). Blast2go was used for Gene Ontology (GO) annotation, and hmmer was used for Pfam annotation. *Dictyophora rubrovolvata* gene models were aligned to fungal cytochrome P450 sequences (CYPs), and the detected CYPs were named according to Cytochrome P450 Engineering Database (https://cyped.biocatnet.de). Annotation of carbohydrate-active enzymes (CAZYmes) in *D. rubrovolvata* genomes was carried out by BLASTP analysis against the CAZy database (https://bcb.unl.edu/dbCAN2).

### Other basidiomycete genome sources

Together with *D. rubrovolvata*, 18 other fungal species (*A. bisporus*, *Agrocybe pediades*, *Antrodia cinnamomea*, *Boletus edulis*, *Clathrus columnatus*, *Coprinopsis cinerea*, *Cordyceps militaris*, *Dictyophora indusiata*, *D. rubrovolvata*, *Laccaria bicolor*, *L. edodes*, *Lepista nuda*, *O. sinensis*, *P. eryngii*, *Pleurotus ostreatus*, *Pleurotus pulmonarius*, *S. latifolia*, *Tricholoma matsutake*, *Wolfiporia cocos*) were selected for CAZyme composition analysis. The protein sequences used in the present study were downloaded from the NCBI (National Center for Biotechnology Information, https://www.ncbi.nlm.nih.gov/genome, accessed on: 2022 Nov 18).

## Results and discussion

### Sequencing and assembly data

The final *D. rubrovolvata* genome was composed of 136 contigs after genome assembly. The total length of all assembled contigs was 32,887,457 bp with a GC content of 45.16% and an N50 value of 2,480,000 bp. There were 233 complete BUSCOs in the assembled genes of *D. rubrovolvata*, the complete single-copy BUSCO was 232 (80%), and the complete duplicated BUSCO was 1 (0.34%). The complete BUSCO in *D. rubrovolvata* is lower than those in *D. indusiata*, *S. latifolia*, *Tremella fuciformis*, *Naematelia encephala*, and *G. lucidum* (Supplementary Table 1). The assembly encodes 9,725 protein-coding genes, which is less than the other 16 fungi except *C. militaris* (9,651 protein-coding genes) and *O. sinensis* (6,972 protein-coding genes). The GC content of *D. rubrovolvata* (45.2%) was also lower than the average value of 19 fungi in this study (Supplementary Table 2). The general features of the *D. rubrovolvata* genome, including assembly and gene model statistics, are presented in Table 1.

**Table 1.** General features of the *D. rubrovolvata* genome.

Feature		
*Genome assembly*		
Sequence and assembly statistic	Scaffold length (bp)	32,887,857
	Scaffold number	132
	Scaffold N50 length (bp)	2,711,256
	Scaffold N90 length (bp)	840,000
	Contig length	32,887,457
	Contigs number	136
	Contig N50 length (bp)	2,480,000
	Contig N90 length (bp)	840,000
	GC content (%)	45.16
*Gene prediction*		
Gene	Number of protein-coding genes	9,725
	Total gene length (bp)	20,763,569
	Average gene length (bp)	2,135.07
Exon	Total exon length (bp)	16,349,310
	Average exon length (bp)	237.56
	Total exon number	68,822
	Average number/gene	7.08
CDS	Total CDS length (bp)	14,344,617
	Average CDS length (bp)	215.64
	Total CDS number	66,521
	Average number/gene	6.84
Intron	Total intron length (bp)	4,414,259
	Average intron length (bp)	74.7
	Total intron number	59,097
	Average number/gene	6.08

### Hi-C

Hi-C has been widely used to map chromatin interactions within regions of interest and across the genome. In total, 20.1 million read pairs (6.03 Gb clean data) were generated from the Hi-C library, and the GC content and Q30 ratio (the percentage of clean reads more than 30 bp) were 43.25 and 94.03%, respectively (Supplementary Table 3).

The Hi-C library quality was assessed based on the ratio of mapped reads and the proportions of valid interaction pairs and invalid interaction pairs. Only valid interaction pairs can provide effective information for genome assembly. Invalid interaction pairs mainly consist of self-circle ligation, dangling ends, re-ligation, and dumped pairs. The mapped reads ratio was 95.19% (Supplementary Table 4). Of the unique mapped read pairs, 74.34% were the valid interaction pairs (12.12 million), which were used for the next Hi-C assembly (Supplementary Table 5).

Overall, we constructed a chromosomal-level assembly of *D. rubrovolvata* with 11 pseudo-chromosomes with lengths ranging from 1.77 to 3.37 Mb (Table 2). Hi-C assembly incorporated 28,241,566 bp genomic sequences, accounting for 85.87% of the total sequence length on the chromosomes. The detailed distribution of each chromosome sequence is summarized in Table 2.

**Table 2.** Detailed results of chromosome-level scaffolding using Hi-C technology.

Chromosome	Sequence number	Sequence length (bp)
Chr1	2	3,366,512
Chr2	1	3,149,261
Chr3	3	3,126,176
Chr4	1	2,788,612
Chr5	1	2,754,995
Chr6	1	2,711,256
Chr7	1	2,430,384
Chr8	2	2,398,327
Chr9	1	1,936,385
Chr10	1	1,814,427
Chr11	1	1,765,231
Total sequences clustered (ratio %)	15 (11.03)	28,241,566 (85.87)
Total sequences ordered and oriented (ratio %)	15 (100)	28,241,566 (100)

**Fig. 1.**
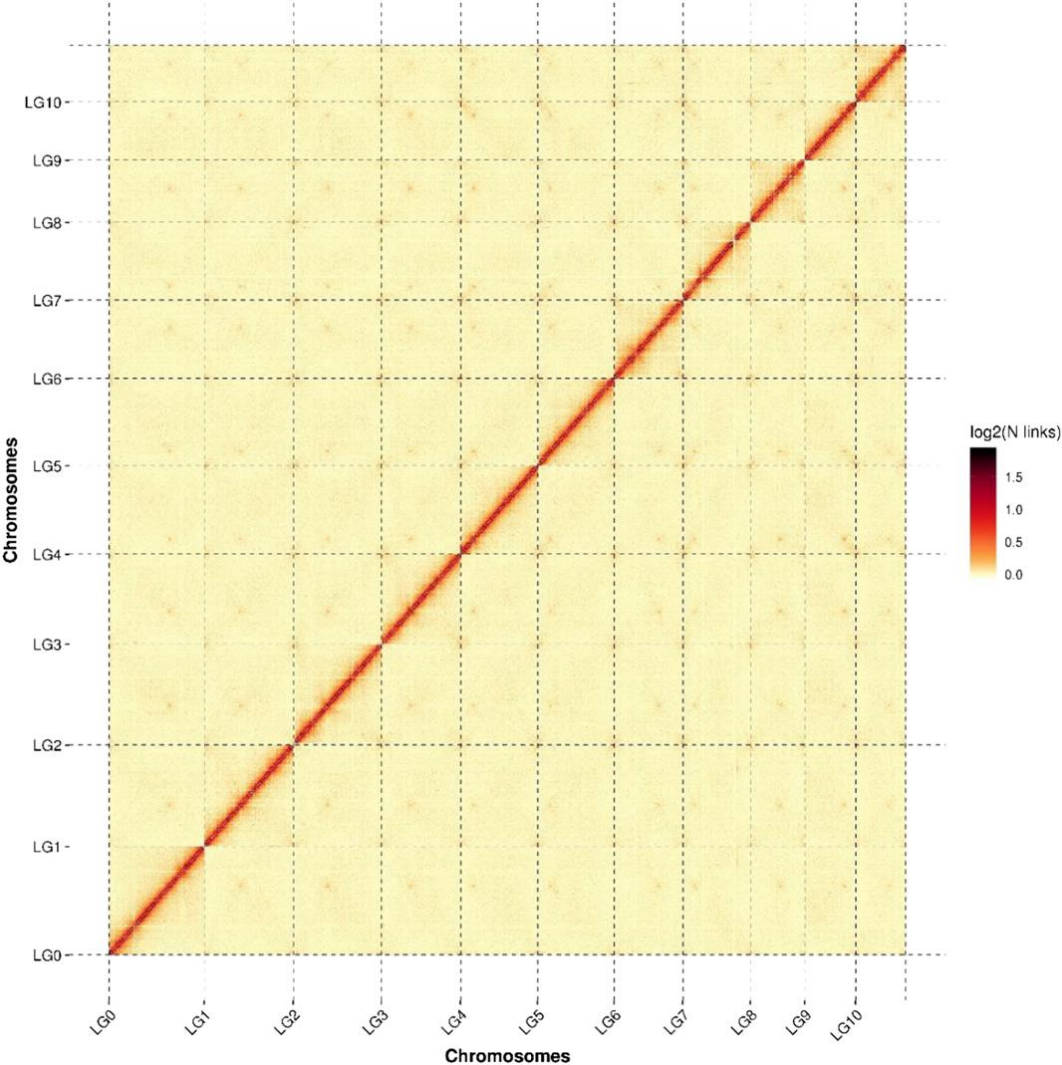
Hi-C assembly of a chromosome interactive heat map. Lachesis Group (LG) means chromosome. LG01–LG10 are the abbreviations of 11 chromosomes. The abscissa and ordinate represent the order of each bin on the corresponding chromosome group.

For the Hi-C assembled chromosomes, the genome was cut into 20 kb bins of equal length. The number of Hi-C read pairs covered between any 2 bins was then used as the intensity signal of the interaction between the bins to construct a heat map (Yang *et al.* 2021). The heat map demonstrated that the 11 chromosome groups can be clearly distinguished (Fig. 1). Within each group, the intensity of interaction in the diagonal position was higher than that in the off-diagonal position, indicating that the intensity of adjacent sequences (diagonal position) interaction in the Hi-C assembly was high, while the intensity of nonadjacent sequences (off-diagonal position) interaction was weak. The heat map of the Hi-C assembly interaction bins was consistent with a genome assembly of excellent quality.

### Repeat sequence

The total length of the repeat sequence was 3,243,445 bp, which accounted for 9.86% of the *D. rubrovolvata* genome length. It was subdivided into 5 major types: retrotransposon, transposon, potential host gene, simple sequence repeat (SSR), and unknown duplications. A total of 2,428 retrotransposon, 2,141,399 bp in length, accounted for 6.51% of the genome length. In retrotransposon, the long terminal repeat-retrotransposons Copia (LTR/Copia) and long terminal repeat-retrotransposons Gypsy (LTR/Gypsy) accounted for 0.49 and 2.68% of the assembled genome, respectively. Transposon represented 0.71% of the assembled genomes. The Helitron transposable element, miniature inverted repeat transposable element, and terminal inverted repeat transposable element accounted for 0.16, 0.09, and 0.38% of the assembled genome, respectively (Table 3).

**Table 3.** Statistical results of genomic repeat sequencing.

Type	Number	Length (bp)	Percentage
Retrotransposon	2,428	2,141,399	6.51
Retrotransposon/DIRS	1	53	0.00
Retrotransposon/LINE	35	2,635	0.01
Retrotransposon/LTR/Copia	264	159,709	0.49
Retrotransposon/LTR/Gypsy	1,303	880,309	2.68
Retrotransposon/PLE|LARD	824	1,125,981	3.42
Retrotransposon/unknown	1	68	0.00
Transposon	526	233,980	0.71
Transposon/Helitron	127	51,911	0.16
Transposon/MITE	147	30,361	0.09
Transposon/TIR	224	125,860	0.38
Transposon/unknown	28	26,234	0.08
Potential host gene	226	210,983	0.64
SSR	11	1,006	0.00
Unknown	1,772	740,122	2.25

### Noncoding RNA

The results of noncoding RNAs in the *D. rubrovolvata* Di001 genome were shown in Table 4. With regard to RNA, 329 rRNAs, 150 tRNAs, and 29 other ncRNAs were predicted. Among the rRNAs, there were 79 5S_rRNA, 82 5.8S_rRNA, 88 18S_rRNA, and 80 28S_rRNA.

**Table 4.** Statistical results of noncoding RNAs.

Type		Number
tRNA		150
rRNA	5S_rRNA	79
	5.8S_rRNA	82
	18S_rRNA	88
	28S_rRNA	80
Other ncRNA	U2	6
	RNaseP_nuc	2
	U4	2
	U5	3
	U6	4
	RNase_MRP	1
	Bacteria_small_SRP	1
	Cobalamin	1
	snosnR60_Z15	2
	snoZ13_snr52	1
	snosnR61	1
	Glycine	1
	suhB	1
	snR75	1
	snR56	1
	5_ureB_sRNA	1

### Gene prediction and genome comparisons

**Fig. 2.**
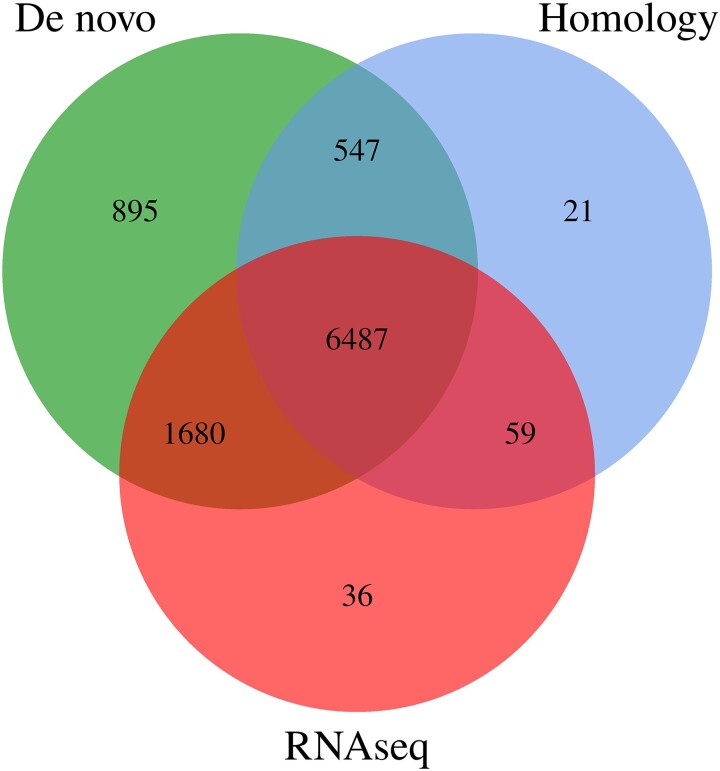
The integrated genes were derived from the statistical plots of 3 predictive methods.

A total of 9,725 genes were predicted in the *D. rubrovolvata* genome (Supplementary Table 6), among which there were 8,830 homology-predicted genes or RNA-seq-predicted genes (90.79%; Fig. 2), indicating high reliability of the prediction. The total length of the encoded genes was 20.76 Mb, accounting for 63.1% of the whole genome, and the average length of each gene was 2,135.07 bp. The average exon and intron numbers were 7.08 and 6.08, respectively (Table 1).

### Gene function annotation

To predict the protein sequences, a similarity analysis of 9,725 nonredundant genes in multiple public databases (GO, KEGG, KOG, NR, Pfam, CAZy, Swiss-Prot, and TrEMBL) identified 8,727 genes that were functionally annotated, which accounted for 89.74% of the assembled genome. Most genes were matched using the Nr (8,671 genes) database, followed by TrEMBL (8,298 genes) and Pfam (6,492 genes) database (Supplementary Table 7).

### KOG annotations

**Fig. 3.**
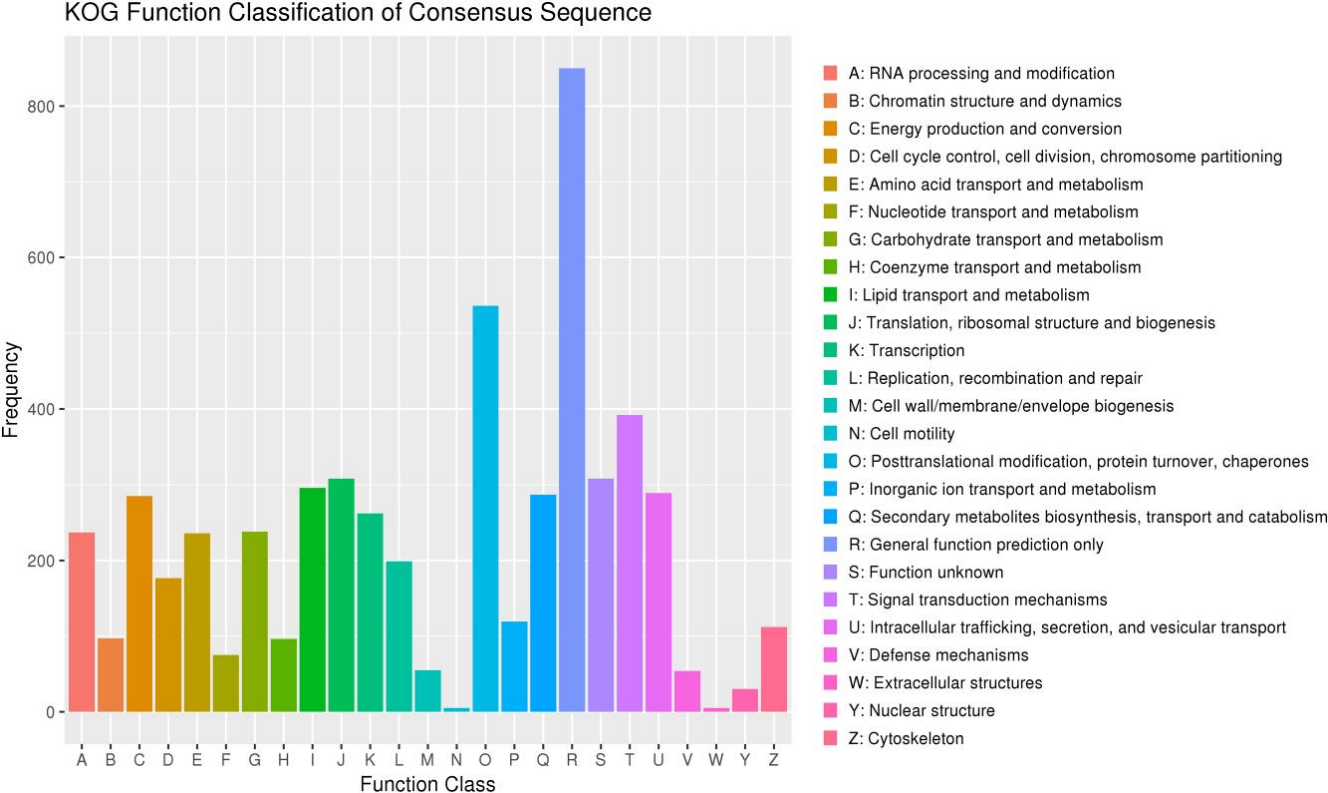
The KOG function classification of proteins.

A statistical map of annotated genes in the KOG database is shown in Fig. 3. A total of 4,899 genes were assigned to 25 categories of KOG, of which the top 5 were “General function prediction only” (850, 15.32%), “Posttranslational modification, protein turnover, chaperones” (536, 9.66%), “Signal transduction mechanisms” (392, 7.07%), “Translation, ribosomal structure and biogenesis” (308, 5.55%), and “Function unknown” (308, 5.55%; Supplementary Table 8).

### GO annotations

**Fig. 4.**
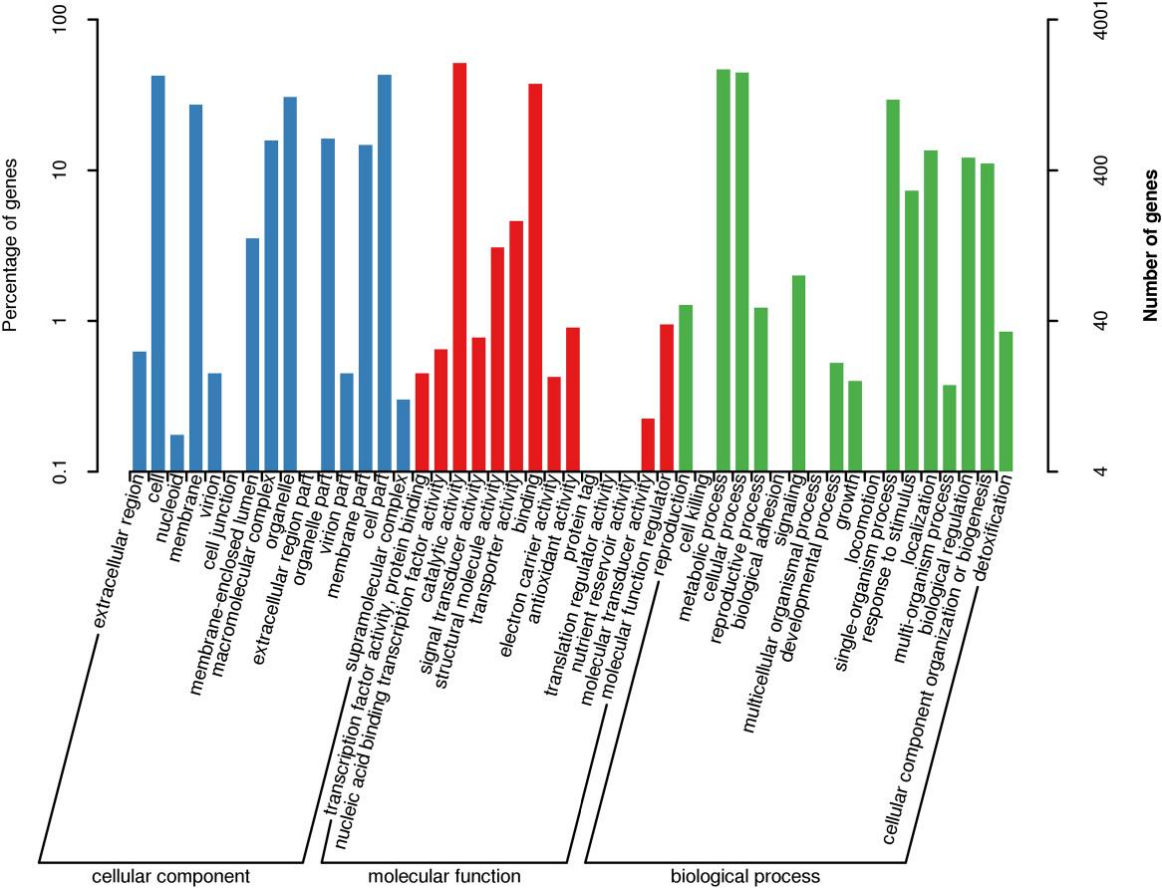
The GO function annotation.

In GO database, 3 independent ontologies including biological process, cellular component, and molecular function were used to describe gene products according to their functional annotations. A total of 4,001 genes were assigned to 3 major categories: biological processes (18 branches), cellular components (15 branches), and molecular functions (14 branches). These were mainly distributed in 5 functional entries, “catalytic activity,” “metabolic process,” “cellular process,” “cell part,” and “cell,” of which the number of annotated genes was 2,058, 1,873, 1,777, 1,722, and 1,702, respectively (Fig. 4). *Dictyophora rubrovolvata* had more genes in the common subcategories of “metabolic process” and “cellular process” within the biological process and “catalytic activity” within the molecular function categories (Supplementary Table 9).

### KEGG annotations

**Fig. 5.**
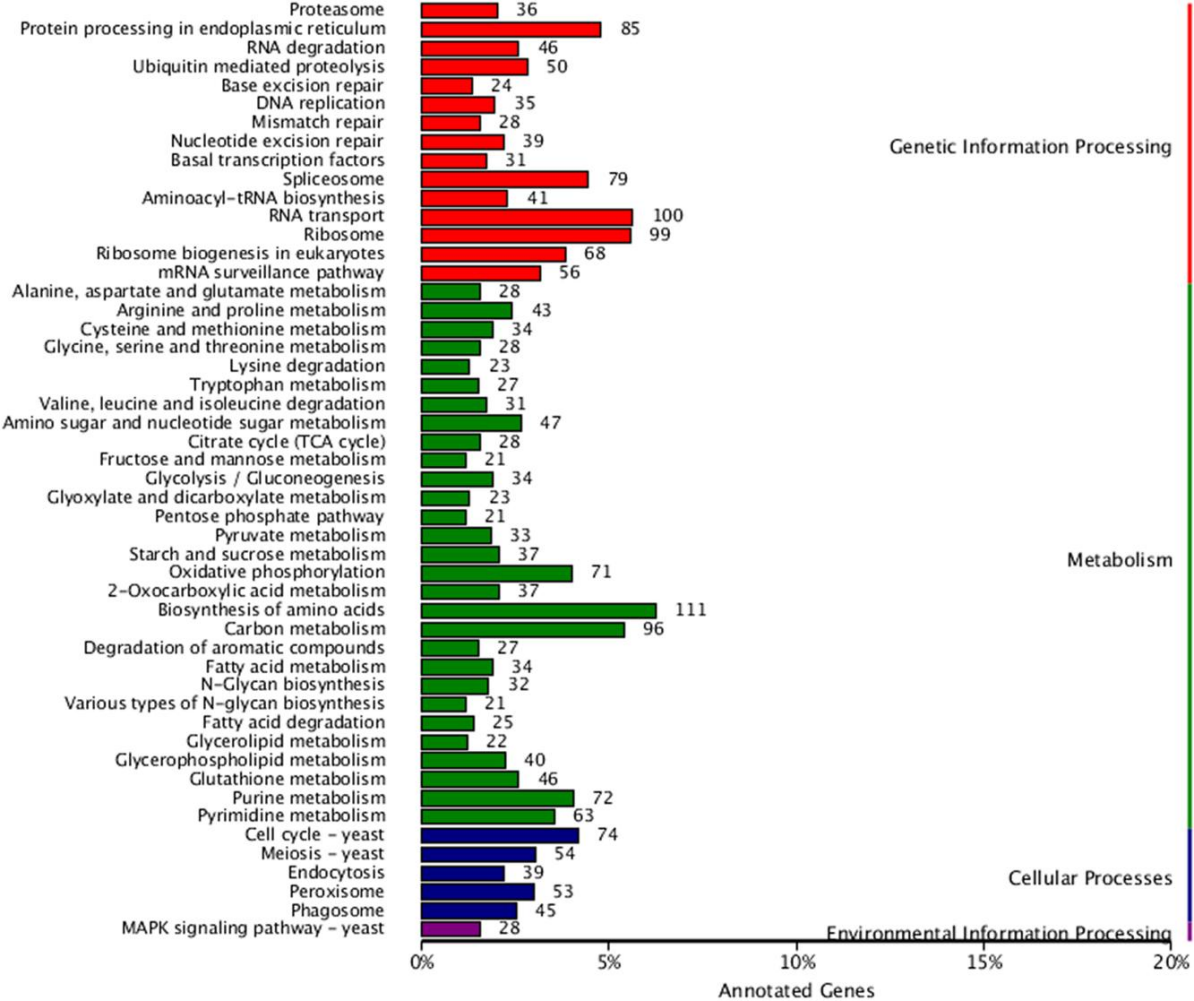
The KEGG function annotation.

To further systematically analyze the metabolic pathways of gene products in cells and the functions of these gene products, the KEGG database was used to annotate the gene functions of *D. rubrovolvata*. A statistical map of the number of annotated genes in the KEGG database is shown in Fig. 5. The 3,046 genes were assigned into 4 categories in KEGG: metabolism (90 branches), genetic information processing (15 branches), cellular processes (5 branches), and environmental information processing (1 branches). Of these, 1,863 genes were assigned to the “metabolism” category. Within metabolism, the biosynthesis of unsaturated fatty acids possesses 111 genes, followed by carbon metabolism (96), amino sugar and nucleotide sugar metabolism (47), and glutathione metabolism (46). A total of 817 genes were assigned to the “genetic information processing” functional category, including nucleocytoplasmic transport (100), ribosome (99), protein processing in the endoplasmic reticulum (85), and spliceosome (79). For cellular processes (265 genes), the cell cycle was the most involved (74). In addition to the above 3 major categories, only 28 genes were assigned to the “environmental information processing” category (Supplementary Table 10).

### CAZymes

**Fig. 6.**
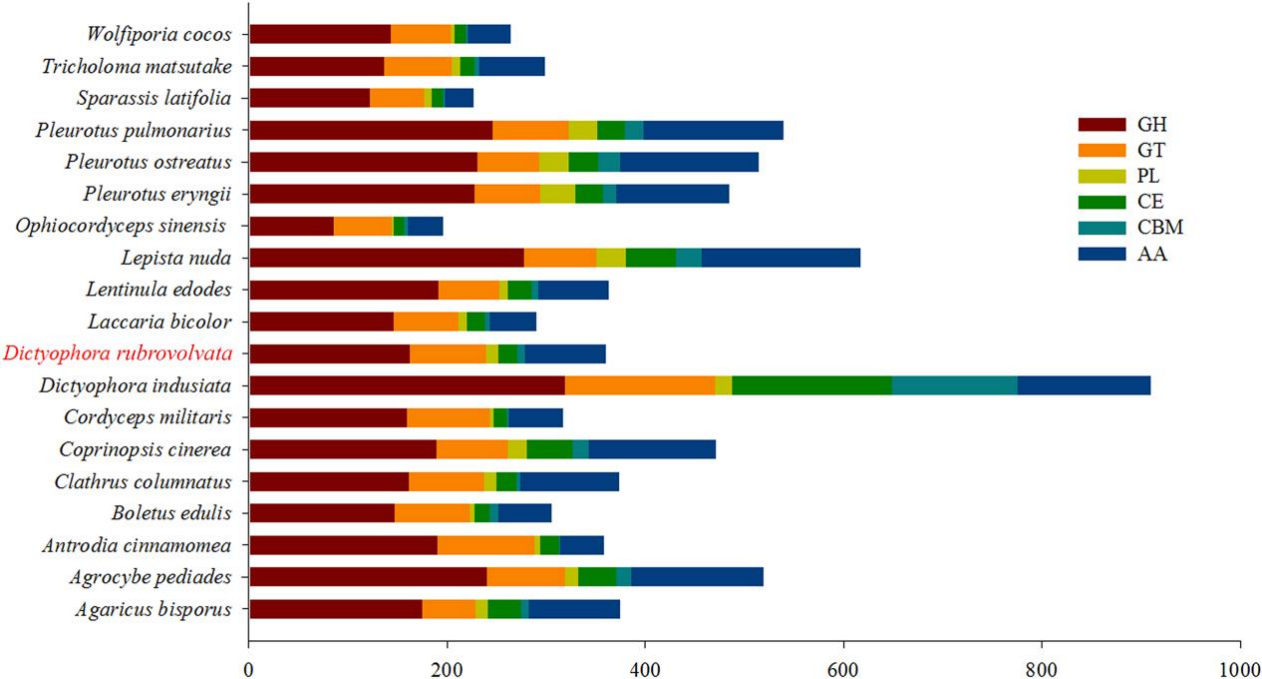
The number of CAZymes genes in *D. rubrovolvata* and the other 18 fungi.

Fungi secrete an array of CAZymes (carbohydrate-active enzymes) and lignin-degrading enzymes for the degradation of lignocellulose. In this study, the CAZymes of *D. rubrovolvata* and 18 other fungi were analyzed (Fig. 6 and Supplementary Table 10). A total of 360 genes were annotated, including 162 glycoside hydrolases (GHs), 77 glycosyl transferases, 12 polysaccharide lyases, 20 carbohydrate esterases (CEs), 7 carbohydrate-binding modules, and 82 auxiliary activities (AAs; Fig. 5 and Supplementary Table 11).

*Dictyophora rubrovolvata* genome had 82 AAs, which more than *A. cinnamomea* (44), *B. edulis* (54), *C. militaris* (55), *L. bicolor* (47), *O. sinensis* (36), *S. latifolia* (38), *T. matsutake* (66), and *W. cocos* (43). Proteins in the AA category were mainly distributed in AA3 (28), AA7 (16), AA1 (11), AA2 (7), AA9 (7), and AA5 (5). GHs accounted for 45% of the total identified CAZymes in *D. rubrovolvata*. 28 genes (18 in GH5 and 10 in GH3) were identified in *D. rubrovolvata*, and these genes were related to cellulose digestion. Twenty-five genes (18 in GH16, 4 in GH43, and 3 in GH10) were also identified, and these genes were involved in hemicellulose digestion. Proteins in the CE category were mainly distributed in CE16 (6, 30%), CE17 (4, 20%), CE4 (3, 15%), and CE8 (2, 10%).

### The CYPs family

The CYP superfamily is a diverse group of enzymes involved in various physiological processes, including detoxification, degradation of xenobiotics, and the biosynthesis of secondary metabolites (Yap *et al.* 2014). *Dictyophora rubrovolvata* had a total of 425 CYP genes, which can be classified into 41 families according to Nelson's nomenclature (Fischer *et al.* 2007). The CYP51 family, which may play a role in demethylation, was found to have the greatest number of genes (118 genes, 27.8%), followed by CYP 620 family (52 genes, 12.2%), CYP53 family (43 genes, 10.1%), and CYP 504 family (27 genes, 6.4%). Proteins in the CYP51 family were mainly consisted of CYP51_422 (33 genes, 28.0%), CYP51_6 (31 genes, 26.3%), and CYP51_11 (14, 11.9%; Supplementary Table 12).

### Conclusion

In this study, we report a highly accurate chromosome-level genome assembly of *D. rubrovolvata* based on the PacBio SMRT and Hi-C technologies. The final genome size was 32.89 Mb. A total of 9,725 protein-coding genes were predicted using the strategy of multievidence combination, and 8,727 genes were functionally annotated. To the best of our knowledge, this genome-wide assembly and annotation data represent the first genome scale assembly of *D. rubrovolvata*. The genome data created in this study will serve as valuable resources for fungal diversity research and breeding of *D. rubrovolvata* and will further provide essential genomic information for understanding the molecular mechanism in its fruiting body formation during morphological development and facilitate the exploitation of medicinal compounds produced by this mushroom.

## Supplementary Material

jkad102_Supplementary_Data

## Data Availability

Genome sequencing of *D. rubrovolvata* Di001 generated for this study has been submitted to the NCBI (BioProject: PRJNA908074 and BioSample: SAMN32024313). Supplemental material available at G3 online.

## References

[jkad102-B1] Alioto T, Blanco E, Parra G, Guigó R. Using geneid to identify genes. Curr Protoc Bioinformatics. 2018;64(1):e56. doi:10.1002/cpbi.56. 30332532

[jkad102-B2] Bao W, Kojima KK, Kohany O. Repbase update, a database of repetitive elements in eukaryotic genomes. Mob DNA. 2015;6(1):11. doi:10.1186/s13100-015-0041-9. 26045719 PMC4455052

[jkad102-B3] Belton JM, McCord RP, Gibcus J, Naumova N, Zhan Y, Dekker J. Hi-C: a comprehensive technique to capture the conformation of genomes. Methods. 2012;58(3):268–276. doi:10.1016/j.ymeth.2012.05.001. 22652625 PMC3874846

[jkad102-B4] Berlin K, Koren S, Chin CS, Drake J, Landolin JM, Phillippy AM. Assembling large genomes with single-molecule sequencing and locality-sensitive hashing. Nat Biotechnol. 2015;33(6):623–630. doi:10.1038/nbt.3238. 26006009

[jkad102-B5] Burge C, Karlin S. Prediction of complete gene structures in human genomic DNA. J Mol Biol. 1997;268(1):78–94. doi:10.1006/jmbi.1997.0951. 9149143

[jkad102-B6] Burton JN, Adey A, Patwardhan RP, Qiu R, Kitzman JO, Shendure J. Chromosome-scale scaffolding of de novo genome assemblies based on chromatin interactions. Nat Biotechnol. 2013;31(12):1119–1125. doi:10.1038/nbt.2727. 24185095 PMC4117202

[jkad102-B7] Campbell MA, Haas BJ, Hamilton JP, Mount SM, Buell CR. Comprehensive analysis of alternative splicing in rice and comparative analyses with Arabidopsis. BMC Genomics. 2006;7(1):327. doi:10.1186/1471-2164-7-327. 17194304 PMC1769492

[jkad102-B8] Cheng H, Concepcion GT, Feng X, Zhang H, Li H. Haplotype-resolved de novo assembly using phased assembly graphs with hifiasm. Nat Methods. 2021;18(2):170–175. doi:10.1038/s41592-020-01056-5. 33526886 PMC7961889

[jkad102-B9] Dai YT, Sun L, Yin XL, Gao M, Zhao YT, Jia PS, Yuan XH, Fu YP, Li Y. *Pleurotus eryngii* genomes reveal evolution and adaptation to the Gobi Desert environment. Front Microbiol. 2019;10:2024–2035. doi:10.3389/fmicb.2019.02024. 31551962 PMC6734163

[jkad102-B10] Deng C, Shang JY, Fu HT, Chen JX, Liu HY, Chen JH. Mechanism of the immunostimulatory activity by a polysaccharide from *Dictyophora indusiata*. Int J Biol Macromol. 2016;91:752–759. doi:10.1016/j.ijbiomac.2016.06.024. 27293036

[jkad102-B11] Edgar RC, Myers EW. PILER: identification and classification of genomic repeats. Bioinformatics. 2005;21(Suppl. 1):i152–i158. doi:10.1093/bioinformatics/bti1003. 15961452

[jkad102-B12] Fischer M, Knoll M, Sirim D, Wagner F, Funke S, Pleiss J. The cytochrome P450 engineering database: a navigation and prediction tool for the cytochrome P450 protein family. Bioinformatics. 2007;23(15):2015–2017. doi:10.1093/bioinformatics/btm268. 17510166

[jkad102-B13] Fu Y, Lin S, Lu M, Wei SY, Zhou J, Zhao L, Zhang Q, Lin DR, Liu YT, Chen H, et al Quantitative evaluation of ultrasound-assisted extraction of 1,3-β-glucans from *Dictyophora indusiata* using an improved fluorometric assay. Polymers (Basel). 2019;11(5):864. doi:10.3390/polym11050864. 31086008 PMC6572555

[jkad102-B14] Gao Q, Yan D, Song S, Fan YY, Wang SX, Liu Y, Huang Y, Rong CB, Guo Y, Zhao S, et al Haplotype-resolved genome analyses reveal genetically distinct nuclei within a commercial cultivar of *Lentinula edodes*. J Fungi (Basel). 2022;8(2):167. doi:10.3390/jof8020167. 35205921 PMC8877449

[jkad102-B15] Haas BJ, Salzberg SL, Zhu W, Pertea M, Allen JE, Orvis J, White O, Buell CR, Wortman JR. Automated eukaryotic gene structure annotation using EVidenceModeler and the program to assemble spliced alignments. Genome Biol. 2008;9(1):R7. doi:10.1186/gb-2008-9-1-r7. 18190707 PMC2395244

[jkad102-B16] Han YJ, Wessler SR. MITE-hunter: a program for discovering miniature inverted-repeat transposable elements from genomic sequences. Nucleic Acids Res. 2010;38(22):e199. doi:10.1093/nar/gkq862. 20880995 PMC3001096

[jkad102-B17] Hang MQ, Zou QQ, Tian HY, Sun BG, Chen HT. Analysis of volatile components from *Dictyophora rubrovolota Zang, Ji et liou*. Procedia Eng. 2012;37:240–249. doi:10.1016/j.proeng.2012.04.234.

[jkad102-B18] He K, Zhao L, Yuan Z, Canario A, Liu Q, Chen S, Guo J, Luo W, Yan H, Zhang D, et al Chromosome-level genome assembly of largemouth bass (*Micropterus salmoides*) using PacBio and Hi-C technologies. Sci Data. 2022;9(1):482. doi:10.1038/s41597-022-01601-1. 35933561 PMC9357066

[jkad102-B19] Jiang JH, Wu SH, Zhou LW. The first whole genome sequencing of *Sanghuangporus sanghuang* provides insights into its medicinal application and evolution. J Fungi (Basel). 2021;7(10):787. doi:10.3390/jof7100787. 34682209 PMC8537844

[jkad102-B20] Jin Jing Z, Ang R, Hui C, Ming Wen Z, Liang S, Ming Jie C, Hong W, Zhi Yong F. Transcriptome analysis and its application in identifying genes associated with fruiting body development in basidiomycete *Hypsizygus marmoreus*. PLoS One. 2015;10(4):e0123025. doi:10.1371/journal.pone.0123025. PMC4383556 25837428

[jkad102-B21] Keilwagen J, Wenk M, Erickson JL, Schattat MH, Grau J, Hartung F. Using intron position conservation for homology-based gene prediction. Nucleic Acids Res. 2016;44(9):e89. doi:10.1093/nar/gkw092. 26893356 PMC4872089

[jkad102-B22] Korf I . Gene finding in novel genomes. BMC Bioinformatics. 2004;5:59. doi:10.1186/1471-2105-5-59. 15144565 PMC421630

[jkad102-B23] Li S, Zhao SZ, Hu C, Mao C, Guo L, Yu H, Yu H. Whole genome sequence of an edible mushroom *Stropharia rugosoannulata* (Daqiugaigu). J Fungi (Basel). 2022;8(2):99. doi:10.3390/jof8020099. 35205854 PMC8880121

[jkad102-B24] Liao WZ, Luo Z, Liu D, Ning ZX, Yang J, Ren JY. Structure characterization of a novel polysaccharide from *Dictyophora indusiata* and its macrophage immunomodulatory activities. J Agric Food Chem. 2015;63(2):535–544. doi:10.1021/jf504677r. 25525995

[jkad102-B25] Majoros WH, Pertea M, Salzberg SL. Tigrscan and GlimmerHMM: two open source ab initio eukaryotic gene-finders. Bioinformatics. 2004;20(16):2878–2879. doi:10.1093/bioinformatics/bth315. 15145805

[jkad102-B26] Meng GL, Du Z, Zhang XL, Yang RH, Wang YH, Lu JJ, Yu D, Dong CH, Yao YJ, Li Y. A preliminary study of chromosome number in *Ophiocordyceps sinensis*. Chin J Appl Entomol. 2021;58(6):1330–1338. doi:10.7679/j.issn.2095-1353.2021.133.

[jkad102-B27] Morin E, Kohler A, Baker AR, Foulongne-Oriol M, Lombard V, Nagy LG, Ohm RA, Patyshakuliyeva A, Brun A, Aerts AL, et al Genome sequence of the button mushroom *Agaricus bisporus* reveals mechanisms governing adaptation to a humic-rich ecological niche. Proc Natl Acad Sci U S A. 2012;109(43):17501–17506. doi:10.1073/pnas.1206847109. 23045686 PMC3491501

[jkad102-B28] Park YJ, Baek JH, Lee S, Kim C, Rhee H, Kim H, Seo JS, Park HR, Yoon DE, Nam JY, et al Whole genome and global gene expression analyses of the model mushroom *Flammulina velutipes* reveal a high capacity for lignocellulose degradation. PLoS One. 2014;9(4):e93560. doi:10.1371/journal.pone.0093560. PMC3979922 24714189

[jkad102-B29] Pertea M, Kim D, Pertea GM, Leek JT, Salzberg SL. Transcript-level expression analysis of RNA-seq experiments with HISAT, StringTie and Ballgown. Nat Protoc. 2016;11(9):1650–1667. doi:10.1038/nprot.2016.095. 27560171 PMC5032908

[jkad102-B30] Price AL, Jones NC, Pevzner PA. De novo identification of repeat families in large genomes. Bioinformatics. 2005;21(Suppl. 1):i351–i358. doi:10.1093/bioinformatics/bti1018. 15961478

[jkad102-B31] Servant N, Varoquaux N, Lajoie BR, Viara E, Chen CJ, Vert JP, Heard E, Dekker J, Barillot E. HiC-Pro: an optimized and flexible pipeline for Hi-C data processing. Genome Biol. 2015;16:259. doi:10.1186/s13059-015-0831-x. 26619908 PMC4665391

[jkad102-B32] Shim D, Park SG, Kim K, Bae W, Lee GW, Ha BS, Ro HS, Kim M, Ryoo R, Rhee SK, et al Whole genome de novo sequencing and genome annotation of the world popular cultivated edible mushroom, *Lentinula edodes*. J Biotechnol. 2016;223:24–25. doi:10.1016/j.jbiotec.2016.02.032. 26924240

[jkad102-B33] Stanke M, Waack S. Gene prediction with a hidden Markov model and a new intron submodel. Bioinformatics. 2003;19(Suppl. 2):ii215–ii225. doi:10.1093/bioinformatics/btg1080. 14534192

[jkad102-B34] Sun T, Zhang YX, Jiang H, Yang K, Wang SY, Wang R, Li S, Lei P, Xu H, Qiu YB, et al Whole genome sequencing and annotation of *Naematelia aurantialba* (Basidiomycota, edible-medicinal fungi). J Fungi (Basel). 2021;8(1):6. doi:10.3390/jof8010006. 35049946 PMC8777972

[jkad102-B35] Tarailo-Graovac M, Chen NS. Using RepeatMasker to identify repetitive elements in genomic sequences. Curr Protoc Bioinformatics. 2009;Chapter 4:4.10.1–4.10.14. doi:10.1002/0471250953.bi0410s25. 19274634

[jkad102-B36] Walker BJ, Abeel T, Shea T, Priest M, Earl AM. Pilon: an integrated tool for comprehensive microbial variant detection and genome assembly improvement. PLoS One. 2014;9(11):e112963. doi:10.1371/journal.pone.0112963. PMC4237348 25409509

[jkad102-B37] Wang J, Wen X, Yang B, Liu D, Li X, Geng F. De novo transcriptome and proteome analysis of *Dictyophora indusiata* fruiting bodies provides insights into the changes during morphological development. Int J Biol Macromol. 2020;146:875–886. doi:10.1016/j.ijbiomac.2019.09.210. 31726131

[jkad102-B38] Wicker T, Sabot F, Van AH, Bennetzen JL, Capy P, Chalhoub B, Flavell A, Leroy P, Morgante M, Panaud O, et al A unified classification system for eukaryotic transposable elements. Nat Rev Genet. 2007;8(12):973–982. doi:10.1038/nrg2165. 17984973

[jkad102-B39] Wu TH, Cai MJ, Hu HP, Jiao CW, Zhang Z, Liu YC, Chen J, Xiao C, Li XM, Gao X, et al Whole-genome sequencing and transcriptome analysis of *Ganoderma lucidum* strain Yw-1-5 provides new insights into the enhanced effect of tween80 on exopolysaccharide production. J Fungi (Basel). 2022;8(10):1081. doi:10.3390/jof8101081. 36294646 PMC9605614

[jkad102-B40] Xiao DL, Ma L, Yang C, Ying ZH, Jiang XL, Lin YQ. De novo sequencing of a *Sparassis latifolia* genome and its associated comparative analyses. Can J Infect Dis Med Microbiol. 2018;2018:1857170. doi:10.1155/2018/1857170. PMC5845502 29682127

[jkad102-B41] Xu Z, Wang H. LTR_FINDER: an efficient tool for the prediction of full-length LTR retrotransposons. Nucleic Acids Res. 2007;35(Web Server issue):W265–W268. doi:10.1093/nar/gkm286. 17485477 PMC1933203

[jkad102-B42] Yang C, Ma L, Xiao D, Liu X, Jiang X, Ying Z, Lin Y. Chromosome-scale assembly of the *Sparassis latifolia* genome obtained using long-read and Hi-C sequencing. G3 (Bethesda). 2021;11(8):jkab173. doi:10.1093/g3journal/jkab173. PMC8496284 34021320

[jkad102-B43] Yap H-YY, Chooi YH, Firdaus-Raih M, Fung SY, Ng ST, Tan CS, Tan NH. The genome of the tiger milk mushroom, *Lignosus rhinocerotis*, provides insights into the genetic basis of its medicinal properties. BMC Genomics. 2014;15(1):635. doi:10.1186/1471-2164-15-635. 25073817 PMC4129116

[jkad102-B44] Ye M, Wen Z, Huan JZ, Xie YG. Effects of *Dictyophora rubrovalvata* polysaccharide on anti-fatigue and hypoxia endurance in mice. Nat Prod Res Dev. 2016;28:416–419. doi:10.16333/j.1001-6880.2016.3.017.

[jkad102-B45] Ye M, Wen Z, Peng YF, Zhang DG. Effects of *Dictyophora rubrovalvata* polysaccharide on anti-aging and hypoglycemic in mice. Sci Technol Food Industry. 2016;37(7):343–345. doi:10.13386/j.issn1002-0306.2016.07.057.

[jkad102-B46] Yu HL, Zhang LJ, Shang XD, Peng B, Li Y, Xiao SJ, Tan Q, Fu YP. Chromosomal genome and population genetic analyses to reveal genetic architecture, breeding history and genes related to cadmium accumulation in *Lentinula edodes*. BMC Genomics. 2022;23(1):120. doi:10.1186/s12864-022-08325-x. 35144543 PMC8832684

[jkad102-B47] Zang M, Ji DG. Notes on Phallaceae from the eastern Himalayan region of China. Acta Mycologica Sinica. 1985;4(2):109–117. doi:10.13346/j.mycosystema.1985.02.008.

